# Evaluating Bioassays for the Determination of Simvastatin’s Osteogenic Activity: A Systematic Review

**DOI:** 10.3390/jfb16020061

**Published:** 2025-02-11

**Authors:** Lara Steiner Back, Isabella Schönhofen Manso, Mariane Beatriz Sordi, Gabriel Leonardo Magrin, Águedo Aragonês, Ricardo de Souza Magini, Reinhard Gruber, Ariadne Cristiane Cabral Cruz

**Affiliations:** 1 Post-Graduation Program of Dentistry, Center for Education and Research on Dental Implants, Federal University of Santa Catarina, Florianópolis 88053-701, Brazil; lara_back@msn.com (L.S.B.); isabellamanso@yahoo.com.br (I.S.M.); marianesordi@hotmail.com (M.B.S.); gabriel.magrin@ufsc.br (G.L.M.); aguedo@terra.com.br (Á.A.); ricardo.magini@gmail.com (R.d.S.M.); 2 Department of Oral Biology, University Clinic of Dentistry, Medical University of Vienna, 1090 Vienna, Austria; 3 Applied Virology Laboratory, Federal University of Santa Catarina, Florianópolis 88053-701, Brazil

**Keywords:** simvastatin, mesenchymal stromal cell, osteogenesis, osteoblast, mineralization

## Abstract

Objective: Osteogenic differentiation is a complex process, and its analysis requires several biomarkers. Allied with this, there are no standardized bioassays to monitor the activity of simvastatin in osteogenesis in vitro. Therefore, identifying the most efficient and sensitive bioassays may enhance the quality of in vitro studies, bridging the gap with in vivo findings, saving time and resources, and benefiting the community. This systematic review aimed to determine the most efficient bioassay for simvastatin’s osteogenic activity in vitro, in terms of sensitivity. Materials and Methods: In vitro studies evaluating undifferentiated mesenchymal cells treated with simvastatin were considered eligible. References were selected in a two-phase process. Electronic databases and the grey literature were screened up to September 2023. The Office of Health Assessment and Translation (OHAT) tool was used to assess the risk of bias. Certainty in cumulative evidence was evaluated using the Grading of Recommendations, Assessment, Development, and Evaluation (GRADE) criteria. Data were analyzed considering extracellular matrix mineralization, alkaline phosphatase, and the expression of potential target genes, such as bone morphogenetic protein-2 (BMP-2), collagen type I, Runt-related transcription factor 2, osterix, osteocalcin, and osteopontin. Results: Fourteen studies were included. A “probably low” or a “definitely low” risk of bias was assigned to the included studies. The simvastatin concentration ranged from 0.1 nM to 10 µM. Considering a minimum 4-fold increase, simvastatin caused robust mineralization of the extracellular matrix in four studies (4.0-, 4.4-, 5.0-, and 39.5-fold). Moreover, simvastatin substantially increased BMP-2 expression in mesenchymal cells in three studies (4-, 11-, and 19-fold). Conclusion: Therefore, mineralization of the extracellular matrix and BMP-2 expression in mesenchymal cells are the most efficient bioassays for determining the osteogenic activity of simvastatin in vitro (high certainty level). These findings provide a standardized approach that can enhance the reliability and comparability of in vitro studies, bridging the gap with in vivo research and optimizing resources in the field of bone regeneration.

## 1. Introduction

Bone defects caused by trauma, tumors, tooth loss, or periodontal disease are challenging for clinicians. The regeneration of these defects frequently requires bone grafting before dental implant placement and implant-supported rehabilitation [1,2]. Autologous bone grafts have limitations, including the morbidity of the donor area, limited bone availability for harvesting, and difficulty in predicting resorption over time [3]. In turn, immunogenic issues and the risk of disease transmission are limitations concerning allogeneic bone grafts [4]. Regarding xenogeneic grafts, it is imperative to thoroughly remove proteins during the biomaterial processing to mitigate the risk of immunologic complications and potential contamination [5]. As an alternative approach, tissue engineering takes advantage of bone substitutes to interact with anabolic signaling molecules and mesenchymal cells [6,7,8]. Strategies in bone tissue engineering necessitate a profound understanding of how mesenchymal cells respond to anabolic signals, with simvastatin emerging as one of the most promising pharmacological compounds.

Simvastatin, a member of the statin family [9], is a 3-hydroxy-3-methylglutaryl coenzyme A (HMG-CoA) reductase inhibitor prescribed to prevent cardiovascular diseases [10]. Besides lowering lipids, Mundy et al. [9] demonstrated that simvastatin exerts a bone anabolic effect using calvaria defect models. This effect is mediated through reductions in isoprenoid intermediates, such as farnesyl pyrophosphate and geranylgeranyl pyrophosphate, which are essential for the prenylation of small GTP-binding proteins like Ras, Rho, and Rac. The inhibition of RhoA, in particular, has been associated with the stimulation of bone morphogenetic protein type 2 (BMP-2) expression. Furthermore, simvastatin influences the Wnt/β-catenin signaling pathway, which interacts synergistically with BMP signaling to promote osteoblast differentiation. These mechanisms collectively enhance the activity of Runx2, a master transcription factor for osteoblast differentiation, further supporting its role in bone regeneration [11]. Additionally, drug delivery systems embedding simvastatin into bone substitutes potentially affect simvastatin release kinetics and activity, and have garnered growing attention from the scientific community in recent years [12,13,14,15,16]. Therefore, biomaterials with different compositions have been proposed as drug delivery systems for simvastatin [12,13,17]. Consequently, several in vitro studies have been performed as preliminary evaluations.

It is important to remember that osteogenic differentiation is a complex process that requires the analyses of several early and late biomarkers, including alkaline phosphatase, bone morphogenetic protein-2 (BMP-2), collagen type I, Runt-related transcription factor 2, osterix, osteonectin, osteocalcin, and osteopontin, as well as extracellular matrix mineralization. The variability and inconsistency of results across bioassays make it challenging to determine which assays are the most reliable and efficient for drawing accurate conclusions regarding osteogenic differentiation. Furthermore, the lack of standardized bioassays to specifically monitor the activity of simvastatin in osteogenesis in vitro adds to this challenge. The purpose of this study is to identify the most efficient and sensitive bioassays to enhance the quality of in vitro studies, bridging the gap with in vivo findings, saving time and resources, and ultimately benefiting the broader scientifical community.

Considering the effect of simvastatin in osteogenesis in vitro, various types of mesenchymal cells have been evaluated, such as osteogenic cell lines [18,19,20], cells from murine (bone marrow-derived mesenchymal cells [15,16,21,22,23,24]), from human (adipose-, bone marrow-, and human amniotic membrane epithelial-derived mesenchymal cells [14,25,26,27]), and embryonic stem cells [28,29]. Based on these cell types, the following outcome variables have previously been evaluated: matrix mineralization [14,15,16,22,23,25,26,27,28,29,30], alkaline phosphatase (ALP) [14,15,16,21,23,24,25,26,27,28,29,30], bone morphogenetic protein type-2 (BMP-2) [14,15,23,24,25], collagen type I (COL1) [16,22,28,29,31], osteocalcin (BGLAP) [14,15,16,23,24,28], Runt-related transcription factor 2 (RUNX2) [14,15,16,21,28,29,31], osterix (OSX) [21,28,29], and osteopontin [24]. As can be seen, there is a large spectrum of mesenchymal cells and outcome parameters to measure simvastatin activity in vitro. Therefore, through a systematic review, we aimed to identify the most sensitive outcome variables to be used as simvastatin bioassays for osteogenesis in vitro. To estimate the x-fold increase in each bioassay induced by simvastatin, data from the simvastatin-treated group were normalized against the control group. The control group could consist of either an osteogenic medium or a non-osteogenic medium, both without simvastatin. A minimum 4-fold increase was considered robust.

## 2. Materials and Methods

### 2.1. Study Design

The systematic review protocol was based on the Cochrane Handbook for Systematic Reviews of Interventions [32] and Preferred Reporting Items for Systematic Review and Meta-Analysis (PRISMA 2020) checklist guidelines [33]. The protocol was registered on the Open Science Framework registration platform (10.17605/OSF.IO/GBK5S) on 27 August 2020.

### 2.2. Inclusion Criteria

The PICOS factors (population, intervention, comparison, outcome, and type of studies) used in this systematic review were as follows: Population (P)—undifferentiated mesenchymal cells. Intervention (I)—simvastatin treatment. Comparison (C)—no simvastatin treatment. Outcome (O)—bioassay efficiency. Type of studies (S)—in vitro studies. The focus question was as follows: “What is the most efficient bioassay to evaluate in vitro simvastatin activity during osteogenesis?” Studies were considered eligible when they evaluated undifferentiated mesenchymal cells treated with simvastatin. No publication time restriction was applied.

### 2.3. Exclusion Criteria

The following exclusion criteria were considered: (1) Studies investigating other cell types, such as differentiated cells. (2) Studies assessing drug or biomaterial associations that do not present separate data for simvastatin alone. (3) Studies that do not present an untreated control group with the same cell type. (4) Studies that do not present a control group without simvastatin treatment. (5) Studies that do not evaluate osteogenic differentiation. (6) Studies that are not in vitro (clinical studies, animal studies, conference abstracts, letters, pilot studies, review articles, case reports, protocols, short communications, personal opinions, posters, and book chapters). (7) Studies that do not specify the culture medium. (8) Studies that do not quantify alkaline phosphatase (ALP) or extracellular matrix mineralization for test and control groups. (9) Full text not available (book chapters, conference abstracts, expert opinions, letters, and literature reviews). (10) Duplicated data (e.g., dissertations/theses in which corresponding published articles were available). (11) Studies not published in the Latin alphabet.

### 2.4. Search Strategy

A search strategy based on the PICOS structure using MeSH terms and keywords was developed. Also, Embase, Literature of Latin American and Caribbean Health Sciences (LILACS), PubMed, SCOPUS, and Web of Science were used as electronic databases. Additionally, the grey literature was consulted with a search strategy for Google Scholar web search (first 100 references) and ProQuest. The electronic search was performed until September 2023 (Appendix A). A hand search on the reference list of identified records was also performed. All records were exported to reference manager software (Mendeley Desktop, Elsevier, London, UK version v1.19.8), and duplicates were removed.

### 2.5. Study Selection

A two-phase selection process was performed. Two independent reviewers (L.S.B. and I.S.M.) selected the references using online software (Rayyan, Qatar Computing Research Institute, Qatar). In phase one, both reviewers read the titles and abstracts independently while applying the eligibility criteria. The reviewers performed a full-text reading using the eligibility criteria in phase two. A third reviewer (M.B.S.) cross-checked the retrieved information in both phases. The final selection was always based on the full-text publication. Articles that met the eligibility criteria proceeded for data extraction.

### 2.6. Data Collection Process and Data Items

Two independent reviewers (L.S.B. and I.S.M.) collected data from the selected articles. Once selected, they cross-checked the retrieved information with a third reviewer (M.B.S.). Any disagreement was discussed among them. The following data were extracted for each included study: author, year of publication, country, cell type, the origin of cell lines, cell treatment, simvastatin concentration, evaluation methods, experimental time, and main results. If data were missing or unclear, we contacted the corresponding authors of the included studies to resolve or clarify the issue.

### 2.7. Risk-of-Bias Assessment in Individual Studies

The Office of Health Assessment and Translation (OHAT) tool was employed to evaluate the risk of bias (RoB) among individual studies [34], with adaptations made to in vitro studies [35]. Briefly, regarding selection bias under the domains “Randomization of the exposure levels” and “Allocation concealment”, all studies using homogeneous cell suspensions were considered as having a definitely low risk of bias. Additionally, confounding bias is not a relevant key item for experimental animal or in vitro studies. Consequently, questions 3 and 4 were not applied. In sequence, two reviewers (L.S.B. and I.S.M.) independently assessed the risk of bias in the included studies. Discrepancies between the reviewers were resolved by discussion until agreement. The possible answers were “definitely low”, “probably low”, “probably high”, or “definitely high” risk of bias, following specific criteria detailed in the protocol. After addressing all the questions, these scores were subsequently categorized into three tiers: Tier 1 (high quality), Tier 2 (moderate quality), or Tier 3 (low quality). Tier 1 is defined as having a “++” (definitely low) or “+” (probably low) risk of bias in all key domains, as well as a “++” (definitely low) or “+” (probably low) risk of bias for ≥50% of the other domains. Tier 2 comprises studies that do not meet the criteria for placement in either Tier 1 or Tier 3. Tier 3 represents a “- -” (definitely high) or “-” (probably high) risk of bias in all key domains, as well as “- -” (definitely high) or “-” (probably high) risk of bias for ≥50% of the other domains.

### 2.8. Summary Measures and Synthesis of Results

Information concerning gene expression analysis (levels of osteogenic markers), bone protein expression, the quantitative staining of extracellular matrix mineralization or calcium, and ALP activity or staining were considered during the evaluation of outcomes. A meta-analysis was planned if the data from the included studies were deemed homogeneous. To estimate the x-fold increase in each bioassay, data from the SIM-treated group were normalized against the SIM control group, which could consist of either an osteogenic medium (OM) or a non-osteogenic medium (NOM) without SIM.

### 2.9. Certainty in Cumulative Evidence and Risk of Bias Across Studies

Methodological heterogeneity was assessed by comparing the variability in study design and the individual RoB of the included studies. The certainty in cumulative evidence was assessed using the Grading of Recommendations, Assessment, Development, and Evaluation (GRADE) criteria [36], considering in vitro studies instead of randomized clinical trials [36]. Two authors (L.S.B. and A.C.C.C) methodically appraised the selected studies, evaluating risk of bias, inconsistency, indirectness, imprecision, and publication bias, and categorized the outcomes across the included articles as having a “high”, “moderate”, “low”, or “very low” quality of evidence, according to the analysis of each study. The specific reasons for each judgment were considered: High certainty—we are very confident that the true effect lies close to that of the estimated effect. Moderate certainty—we are moderately confident in the effect estimate. Therefore, the true effect is likely to be close to the estimate of the effect, but there is a possibility that it is substantially different. Low certainty—our confidence in the estimate is limited. Therefore, the true effect may be substantially different from the estimate of the effect. Very low certainty—we have very little confidence in the effect estimate. The true effect is likely to be substantially different from the estimate of the effect. The following thresholds were considered for overall GRADE assessments: (1) “not serious” if concerns were present in less than 25% of the included studies; (2) “serious” if concerns were present in between 25% and 50% of the included studies; and (3) “very serious” if concerns were present in more than 50% of the included studies. A high level of certainty means the authors have a lot of confidence that the true effect is similar to the estimated effect. Moderate certainty indicates the authors believe the true effect is probably close to the estimated effect. Low certainty suggests the true effect might be markedly different from the estimated effect, while very low certainty indicates the true effect is probably markedly different from the estimated effect.

## 3. Results

### 3.1. Study Selection

**Figure 1:**
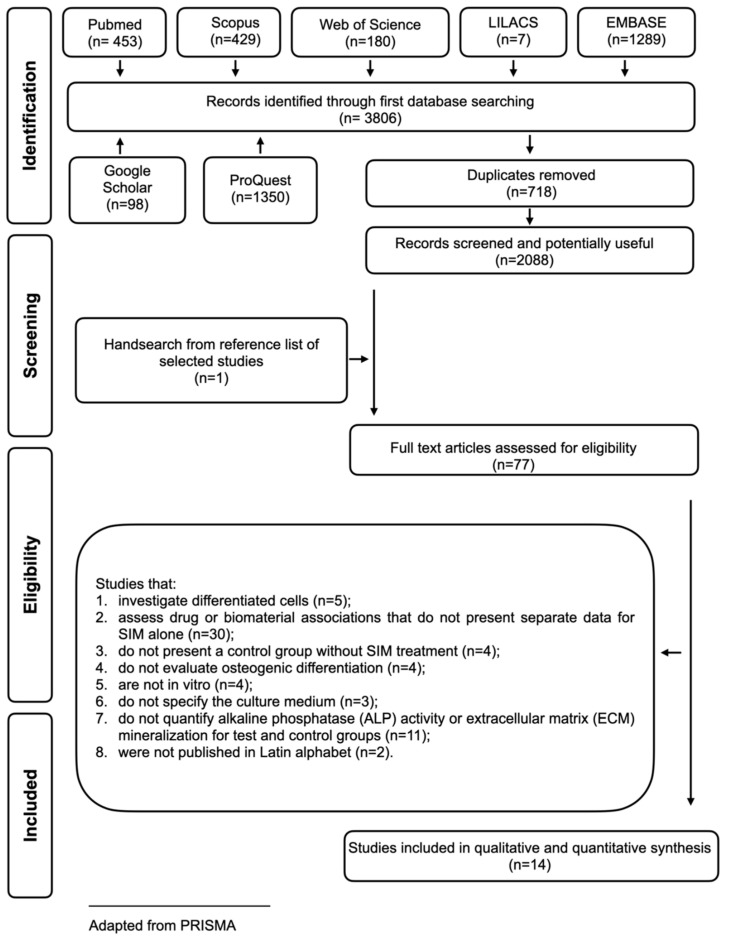
Flow diagram of literature search and selection criteria adapted from PRISMA. References were selected in a two-phase process. Electronic databases (Embase, LILACS, LIVIVO, PubMed, SCOPUS, and Web of Science) and grey literature databases (Google Scholar, Open Grey, and ProQuest) were searched up to September 2023.

In phase one, 2088 records were retrieved from an electronic search of databases and the grey literature after duplicate removal. A comprehensive evaluation of titles and abstracts was performed, resulting in 77 potentially suitable references. After full-text analyses, 14 articles were included in this systematic review according to the inclusion and exclusion criteria. Step-by-step process details are shown in Figure 1. The articles excluded and the reasons for exclusion are detailed in Appendix B.

### 3.2. Study Characteristics

The included studies, published between 2009 and 2023, were conducted in seven countries: China [14,16,21,24,26], Germany [30], South Korea [28,31], Switzerland [25], the United Kingdom [29], Taiwan [15,22,23], and Iran [27].

**Figure 2:**
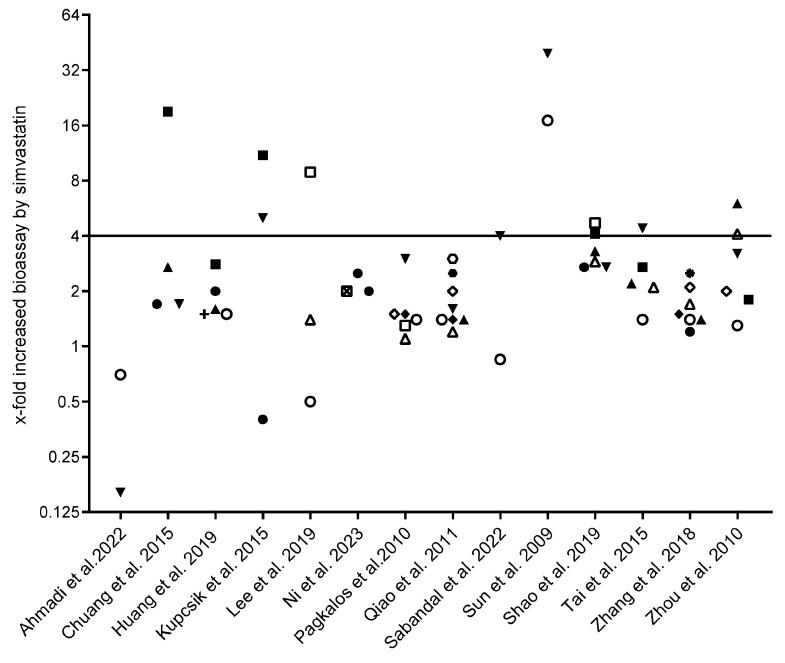
Summary of data showing the x-fold increase determined by each bioassay. The data of the simvastatin-treated group are normalized using the same treatment with no simvastatin (osteogenic medium or non-osteogenic medium). Studies are in alphabetic order. Ahmadi et al. (2022) [27], Chuang et al. (2015) [23], Huang et al. (2019) [24], Kupcsik et al. (2009) [25], Lee et al. (2019) [31], Ni et al. (2023) [16], Pagkalos et al. (2010) [29], Qiao et al. (2011) [28], Sabandal et al. (2022) [30], Shao et al. (2019) [22], Sun et al. (2009) [26], Tai et al. (2015) [15], Zhang et al. (2018) [21], and Zhou et al. (2010) [14]. Each geometric shape represents one bioassay. ■: BMP-2 transcript; ☐: COL1 transcript; ●: ALP transcript; ○: ALP; ▲: BGLAP transcript; △: RUNX2 transcript; ▼: Mineralization; +: BMP-9 transcript; ◆: OSX transcript; ◇: BGLAP; ⊠: IBSP transcript; ✸: RUNX2.

Undifferentiated mesenchymal cell lines from different origins and species were used, including human adipose tissue [14,26], human bone marrow [25], murine bone marrow [15,16,21,23,24], murine embryonic cells [28,29], human gingiva-derived cells [31], and human amniotic membrane epithelial-derived cells [27]. Most studies employed two-dimensional cultures, with one study using three-dimensional spheroids [31]. Cells were treated with non-osteogenic medium (NOM) [14,25,26,29], osteogenic medium (OM) [15,16,21,24,25,26,27,28,29,31], or NOM switching to OM [22,23]. Osteogenic differentiation was evaluated by ALP (activity or histochemical staining), gene expression, extracellular matrix mineralization (Von Kossa staining, Alizarin Red staining, calcium quantification, or phosphorous quantification), and protein expression. Simvastatin molarity was determined. Figure 2 contains the data of each study, showing the x-fold increase determined by each bioassay. The main characteristics of the included studies are provided in Table 1.

### 3.3. Risk of Bias in Individual Studies

Most of the questions were assessed as having a “probably low” or a “definitely low” risk of bias for all included studies. Therefore, as shown in Table 2, all included articles, except one [26], were of high quality (Tier 1) [14,15,16,21,22,23,24,25,26,27,28,29,30,31]. This one article was categorized as Tier 3 [26]. Despite this, all included studies indicated a “probably high” risk of bias for the question regarding the blinding of researchers during the study [14,15,16,21,22,23,24,25,26,27,28,29,30,31]. Blinding minimizes bias by ensuring that researchers are unaware of treatment allocation, which helps prevent conscious or unconscious influences on outcomes. Also, one study [26] did not report the complete outcome data without attrition or exclusion from analysis, and confidence in the outcome assessment was considered to have a “probably high” risk of bias. Additionally, the same study potentially threatened internal validity since the statistical analysis was not described.

### 3.4. Certainty in Cumulative Evidence and Risk of Bias Across Studies

Sources of variability across studies were mostly related to used simvastatin dose and applied methodologies. The certainty in cumulative evidence assessed by modified GRADE criteria was considered low for OPN [28], IBSP [16], and BMP-9 [24]. For ALP [14,15,21,22,23,24,25,26,28,29,30,31], RUNX2 [15,21,22,28,29,31], and OSX [21,28,29], the certainty in cumulative evidence was judged moderate. High certainty in cumulative evidence was addressed to extracellular matrix mineralization [14,15,21,22,23,24,25,26,28,29,30,31], BMP-2 [14,15,23,24,25], COL1 [22,28,29,31], and BGLAP [14,15,21,23,24,28,29]. According to the GRADE tool, the risk of bias across studies was considered as “not serious” since the concerns were present in less than 25% of the included studies. The level of certainty was considered high, meaning that the authors have a lot of confidence that the true effect is similar to the estimated effect. A summary of the findings is presented in Table 3.

### 3.5. Synthesis of Results

Considering a minimum 4-fold increase, as indicated in Figure 2, simvastatin caused a robust extracellular mineralization in four studies (4-, 4.4-, 5-, and 39.5-fold) [15,25,26,30]; two of them compared the effects of simvastatin to the osteogenic medium [15,30], while two compared the simvastatin effect to the non-osteogenic medium [25,26]. BMP-2 expression was at least 4-fold increased by simvastatin in three studies (4.1-, 11-, and 19-fold) [22,24,25]. Occasionally, simvastatin reached a 4-fold increase in COL1 [22,31], BGLAP [14], RUNX2 [14], and ALP activity [26]. Therefore, it is concluded that the extracellular matrix mineralization of mesenchymal cells and BMP-2 expression may be used as the most efficient bioassays for simvastatin in vitro. A high heterogeneity among studies concerning the intervention protocol, applied assays, culture medium, and simvastatin concentration was observed. For instance, simvastatin was tested in the presence [14,15,21,22,23,24,25,26,28,29,31] and absence [14,25,26,29] of osteogenic differentiation medium. The simvastatin concentrations used in the included studies are listed in Table 4. The bioassays evaluated were ALP [15,16,21,22,23,24,25,26,27,28,31], BGLAP [14,15,16,21,22,24], BMP-2 [14,22,23,24], BMP-9 [24], COL1 [16,22,28,29,31], RUNX2 [14,21,28,29,31], OSX [21,28,29], OPN [21], IBSP [16], and ECM mineralization by Alizarin Red [14,15,16,21,22,23,24,28,29], Von Kossa [16,26], and calcium quantification [25,27]. The overall impact of simvastatin on the expression of osteogenic differentiation markers was moderate. A meta-analysis was not conducted due to the heterogeneous data across the included studies. Heterogeneity among studies can significantly impact the data reliability and interpretation and make it challenging to directly compare findings. This variability may lead to inconsistent or conflicting conclusions, reducing the overall confidence.

## 4. Discussion

Simvastatin, embedded within a bone substitute, can support bone regeneration, which requires mesenchymal cell differentiation towards bone-forming osteoblasts. With advances in material engineering, different bone graft compositions have been proposed as simvastatin drug delivery systems [12,13]. Consequently, several preliminary studies evaluating simvastatin for promoting osteogenesis have been performed in vitro [14,22,23,24,31]. However, there is no consensus on which bioassay is more efficient to measure simvastatin activity in vitro. Based on this systematic review, there is accumulating evidence that mineralization of the extracellular matrix and BMP-2 expression are the most efficient bioassays, in terms of sensitivity, to determine the osteogenic activity of simvastatin in vitro. It is relevant to mention that BMP-2 expression, in addition to its sensitivity, is a fast assay due to BMP-2 being an early osteogenic marker. Therefore, the findings of this systematic review may serve as a reference for future studies assessing in vitro osteogenesis promoted by simvastatin.

It has been demonstrated that phosphoinositide 3-kinase (PI3K) contributes to the simvastatin-induced activation of mitogen-activated protein kinases (MAPK), which, together with Akt kinase, regulate BMP-2 expression [38]. The PI3K pathway is activated upon simvastatin treatment, leading to the phosphorylation and activation of Akt (protein kinase B). This activation promotes downstream signaling cascades that enhance the transcriptional activity of BMP-2. The PI3K/Akt pathway also contributes to cell survival and proliferation, which may further support osteogenic differentiation by creating an environment conducive to bone morphogenetic signaling. Similarly, simvastatin influences the MAPK pathway, which involves three main subfamilies: extracellular signal-regulated kinases (ERKs), c-Jun N-terminal kinases (JNKs), and p38 MAPK. Simvastatin has been found to activate the p38 MAPK pathway, which is particularly important for osteogenic differentiation. The activation of p38 MAPK facilitates the phosphorylation of transcription factors, such as Runx2, that interact with the BMP-2 promoter, thereby enhancing BMP-2 gene expression. In some cases, simvastatin also activates ERK1/2, which may act synergistically with p38 to amplify BMP-2 transcription. This information supports BMP-2 as a solid simvastatin target gene. Therefore, these data suggest that BMP-2 is a robust target gene that can complement in vitro mineralization assays to determine simvastatin activity in mesenchymal cells.

We further observed seven studies reporting matrix mineralization. Four studies demonstrated that the mineralization increased approximately 4-fold after 2-3 weeks [14,24,28,30]. In one study, simvastatin increased mineralization after only one week [26]. In support of these observations, simvastatin increased cytosolic and mitochondrial calcium levels in breast cancer cells [39]. Calcium is further linked to BMP-2 expression since extracellular calcium promotes the BMP-2-related osteogenic differentiation of mesenchymal cells [40]. Additionally, simvastatin upregulates Runx2, ALP, osteocalcin, and osteopontin, all of which are critical for matrix maturation and mineralization. Also, simvastatin promotes the activation of the Wnt/β-catenin signaling pathway, which plays a central role in osteoblast differentiation and mineralization [14,21,26,28]. This information could explain why the in vitro mineralization of mesenchymal cells and BMP-2 expression were the most efficient bioassays for simvastatin-induced osteogenic differentiation in vitro. Conversely, less sensitive to simvastatin were the classical markers of osteogenic differentiation, e.g., COL1, BGLAP, RUNX2, and ALP activity performed on day 7 [21,24,28] and day 3 [14,22], as well as after 12 h [15].

Although systematic reviews are designed to answer clinical questions, this review was applied to in vitro studies [41,42]. It is worth mentioning that systematic reviews attempt to identify, appraise, and synthesize all the evidence that meets eligibility criteria to answer a specific research question. Additionally, the systematic review purposes to reveal limitations in the conduct of previous studies that might be addressed in future ones, as well as to propose standardized methods to optimize future studies and allow a comparison among studies [33]. Therefore, this study demonstrated that the mineralization of the extracellular matrix and BMP-2 expression are the most efficient bioassays to determine the osteogenic activity of simvastatin in vitro. Additionally, it was demonstrated that not all the studies mentioned performed this analysis.

Regarding the risk of bias, most questions of the OHAT tool were considered as having a “probably low” or “definitely low” risk of bias. No or minor deviations from the true effect estimation were estimated, providing confidence for interpreting the results [43]. Conversely, all included studies [14,15,16,21,22,23,24,25,26,27,28,29,30,31] failed to report the blinding of the treatment group, which is considered to indicate a “probably high” risk of bias; therefore, conscious or unconscious influences on the outcomes could have occurred. Furthermore, one study [26] presented a “definitely high” risk of bias. Moreover, a high heterogeneity between studies was identified due to the use of different cell lines from murine and human species, as well as different culture conditions and observation periods. All of these may lead to inconsistent or conflicting conclusions and imply that the certainty level of the conclusion is not definitive.

Given the complexity of the osteogenesis process, this research aimed to identify the most efficient bioassay for evaluating in vitro simvastatin activity during osteogenesis. This identification not only aids in data interpretation, but could also suggest additional experiments to enhance comprehensiveness within this topic. It is imperative to underscore that understanding the findings in studies related to in vitro osteogenesis extends beyond the selection of an optimal marker of osteogenic differentiation. Evaluating the specific focus of each study involves not only assessing the outcome, but also understanding the effect of that outcome on osteogenesis as a whole.

Concerning the limitations of this systematic review, the primary aim of this study was to identify the most effective bioassays for assessing the impact of simvastatin on undifferentiated mesenchymal cells during osteogenesis, rather than systematically addressing the variances between different treatments and cell types. However, it was observed that mineralization seems to be an efficient biomarker of osteogenesis for both osteogenic and non-osteogenic media. This was suggested when comparing four studies [15,25,26,30], where, considering a minimum 4-fold increase, simvastatin induced a robust in vitro mineralization of mesenchymal cells. Among these, two studies compared the effects of simvastatin to an osteogenic medium [15,30], while the other two compared simvastatin’s effect to a non-osteogenic medium [25,26]. Regarding BMP-2 expression, two studies used an osteogenic medium [22,24], while one evaluated a non-osteogenic medium [25]. As mentioned, the investigations used undifferentiated mesenchymal cell lines from different origins and species. According to these findings, BMP-2 expression was increased at least 4-fold by simvastatin in three studies, all evaluating bone-marrow-derived cells, irrespective of species (rat [24], mouse [22], or human [25]). Regarding mineralization, two studies focused on bone-marrow-derived mesenchymal cells [15,25], while the other two investigated adipose-derived cells [26,30].

The variability in methodologies among the included studies—such as differences in simvastatin concentrations, cell types, and experimental protocols—represents a limitation for data interpretation in this study. A key purpose of systematic reviews is to identify limitations in previous studies, which can guide future research with the proposal of standardized methods to optimize study designs and enable meaningful comparisons across studies. In this context, it is essential to emphasize that non-immortalized human cell lineages offer a more suitable model for human studies. These cells are human-derived and have not undergone any immortalization processes that might alter their cellular mechanisms. Additionally, if the purpose is to evaluate the osteogenic potential of a substance, it would be more appropriate to use an osteogenic cell culture medium as the control of the differentiation process.

Further investigations are warranted to confirm whether these cells exhibit heightened sensitivity to simvastatin or if the enhancement in BMP-2 expression is specific to these lineages. Hence, additional studies concerning bioassays for determining the simvastatin effect could be conducted, evaluating the influence of different sources of mesenchymal cells and types of cell culture medium. Additionally, relevant information pertaining to high doses of simvastatin and the effect of this on the healing of bone, soft tissue, and cartilage has been published [44,45]. It was not possible to correlate these high doses of simvastatin and their effects with the findings of this review due to the heterogeneity of the cell lineages and methodological protocols previously mentioned. Consequently, future studies are warranted to evaluate the effect of the dose of simvastatin on various human tissues.

## 5. Conclusions

In conclusion, based on a high level of certainty, extracellular matrix mineralization and BMP-2 expression are the most effective bioassays to determine simvastatin’s impact on osteogenesis in vitro. These findings provide a standardized approach that can enhance the reliability and comparability of in vitro studies, bridging the gap with in vivo research and optimizing resources in the field of bone regeneration and tissue engineering applications. However, it is important to consider the high heterogeneity among studies, which may influence the broader applicability of these results. Future studies concerning bioassays determining the effects of simvastatin are warranted in order to evaluate the influence of different sources of mesenchymal cells and types of cell culture medium.

## Figures and Tables

**Table 1:** Summary of descriptive characteristics of included studies (*n* = 14).

Author (Year) Country	Cell Type (Origin)	Treatment Type	SIM Concentration	Evaluation Methods (Period)	mRNA Expression	Western Blotting/ Immunohistochemistry/ Biochemistry	Mineralization
Ahmadi et al. (2022) [27] Iran	Human amniotic epithelial stem cells	T3(o) T4(o) SIM-OM compared to OM	100 nM	ALP activity (Day 14) Calcium quantification (Day 14) Phosphorous quantification (Day 14)	Not evaluated	ALP Activity ↓	Calcium quantification ↓ Phosphorous quantification ↓
Chuang et al., 2015 [23] Taiwan	mBMSCs (Murine bone marrow—D1 Cells)	T5(q) T6(q) SIM-OM compared to OM	0.5 μM	RT-PCR (Days 1, 3, and 5) ALP Staining (Day 5) Alizarin Red staining (5 days after OM induction)	ALP transcript ↑ BGLAP transcript ↑ BMP-2 transcript ↑	ALP ↑ BMP-2 ↑	Alizarin Red staining ↑
Huang et al. (2019) [24] China	mBMSCs (Murine bone marrow)	T3(e) T4(e) SIM-OM compared to OM	0.025, 0.10, 0.25, and 1.0 μM	RT-PCR (Days 3 and 7) ALP activity (Days 3 and 7) Alizarin Red staining (Days 3, 7, and 14)	ALP transcript ↑ BGLAP transcript ↑ BMP-2 transcript ↑ BMP-9 transcript ↑	ALP activity ↑	Alizarin Red staining not quantified
Kupcsik et al. (2009) [25] Switzerland	hBMSCs (Human bone marrow)	T1(a) T2(a) T3(f) SIM-NOM compared to NOM	1 and 5 μM	RT-PCR (Days 4, 11, and 18) ALP activity (Day 11) Von Kossa staining (Day 25) Calcium deposition (Days 18 and 25)	ALP transcript ↓	ALP activity ↓	Calcium quantification ↑ Von Kossa staining not quantified
Lee et al. (2019) [31] South Korea	GMSCs spheroids (Human gingiva)	T3(j) T4(j) SIM-OM compared to OM	1 and 10 μM	RT-PCR (Day 7) ALP activity (Day 14) Alizarin Red staining (Days 7 and 14)	COL1 transcript ↑ RUNX2 transcript ↑	ALP activity ↓	Alizarin Red staining not quantified
Ni et al. (2023) [16] China	mBMSCs (Murine bone marrow)	T3(p) T4(p) SIM-OM compared to OM	100 nM	RT-PCR (Days 7 and 14) ALP staining (Days 7 and 14) Alizarin Red staining( Days 7 and 14) Von Kossa staining (Days 7 and 14)	COL1 transcript ↑ RUNX2 transcript ↑ BGLAP transcript ↑ IBSP transcript ↑	ALP ↑ COL1 ↑ RUNX2 ↑ BGLAP ↑	Alizarin Red staining ↑ Von Kossa staining ↑
Pagkalos et al. (2010) [29] United Kingdom	mESCs (Murine embryonic cells, E14Tg2α)	T1(b) T2(b) T3(g) SIM-NOM compared to NOM	0.1, 1, 10, and 100 nM	RT-PCR (Day 12) ALP activity (Days 4, 8, 12, 16, and 21) Alizarin Red staining (Days 6, 11, 12, 16, and 21)	BGLAP transcript ↑ COL1 transcript ↑ RUNX2 transcript ↑ OSX transcript ↑	ALP ↑	Alizarin Red staining ↑
Qiao et al. (2011) [28] Korea	mESCs (Murine embryonic cells, D3 line ATCC)	T3(h) T4(h) SIM-OM compared to OM	1, 10, 100, 200 nM	RT-PCR (Day 4) ALP activity (Days 4 and 7) Alizarin Red staining (Days 7 and 14) Western blot (Day 7)	BGLAP transcript ↑ COL1 transcript ↑ RUNX2 transcript ↑ OSX transcript ↑	ALP ↑	Alizarin Red staining ↑
Sabandal et al. (2022) [30] Germany	hADSC	T3 (i) T4 (i) SIM-OM compared to OM	0.01, 0.1, 1 and 2 μM	ALP activity (days 18, 21, 28) Alizarin Red staining (days 18, 21, 28)	Not evaluated	ALP ↓	Alizarin Red staining ↑
Shao et al. (2019) [22] Taiwan	mBMSCs (Murine bone marrow, D1 Cells)	T5(r) T6(r) SIM-OM compared to OM	0.1, 0.2 and 0.5 μM	RT-PCR (Day 3) ALP activity (1 day after OM induction—day 4) Alizarin Red staining (5 days after OM induction—day 7) Calcium deposition (5 days after OM induction—day 7)	ALP transcript ↑ BMP2 transcript ↑ COL1 transcript ↑	ALP ↑	Alizarin Red staining ↑
Sun et al. (2009) [26] China	ADSCs (Human adipose tissue)	T1(c) T2(c) T3(k) SIM-NOM compared to NOM	1 μM	RT-PCR (Days 1, 3, 6, 7, 9, and 12) ALP staining (Days 7, 14, 21, and 28) Von kossa staining (Days 7, 14, 21, and 28)	RT-PCR data were not quantified	ALP ↑	Von Kossa staining ↑
Tai et al. (2015) [15] Taiwan	mBMSCs (Murine bone marrow, D1 Cells)	T3(n) T4(n) SIM-OM compared to OM	1 μM	RT-PCR (12, 24, and 48 h) ALP staining (Day 3) Alizarin Red staining (Day 5)	BGLAP transcript ↑ BMP-2 transcript ↑ RUNX2 transcript ↑	ALP ↑	Alizarin Red staining ↑
Zhang et al. (2018) [21] China	mBMSCs (Murine bone marrow)	T3(l) T4(l) SIM-OM compared to OM	0.3 nM	RT-PCR (Day 7) ALP activity (Day 7) ALP staining (Day 7) Alizarin Red staining (Day 7) Western blot (Day 7)	ALP transcript ↑ BGLAP transcript A ↑ RUNX2 transcript ↑ OSX transcript ↑	ALP ↑ BGLAP ↑ OPN ↑ RUNX2 ↑	Alizarin Red staining ↑
Zhou et al. (2010) [14] China	ADSCs (Human adipose tissue)	T1(d) T2(d) T3(m) SIM-NOM compared to NOM	0.01, 0.1, and 1 μM	RT-PCR (Day 3) ALP activity (Days 6 and 14) Alizarin Red staining (Day 14) Osteocalcin radioimmunoassay	BGLAP transcript ↑ BMP2 transcript ↑ RUNX2 transcript A ↑	ALP ↑ BGLAP ↑	Alizarin Red staining ↑

Treatment type composition: T1—non-osteogenic medium (NOM). T2—simvastatin–NOM (SIM-NOM). T3—osteogenic medium (OM). T4—SIM-OM. T5—NOM changing to OM. T6—SIM-NOM changing to OM. NOM: (a) minimum essential medium alfa (α-MEM) + 10% FBS. (b) α-MEM + 15% FBS + 1% penicillin/streptomycin + 10 mM β-glycerophosphate. (c) α-MEM + 10% FBS + 1% penicillin/streptomycin. (d) DMEM + 10% FBS + 100 U/mL penicillin + 100 mg/mL streptomycin. OM: (e) Roswell Park Memorial Institute 1640 Medium (RPMI1640) + 10% FBS + 0.2 mmol/L ascorbic acid + 10 nmol/L dexamethasone + 10 mM β-glycerophosphate. (f) α-MEM + 10% FBS + non-essential amino acids + 10 mmol/L β-glycerophosphate + 0.1 mmol/L ascorbic acid + 10 nmol/L dexamethasone. (g) α-MEM + 15% FBS + 1% penicillin/streptomycin + 10 mM β-glycerophosphate + 50 μg/mL ascorbic acid + 1 μM dexamethasone. (h) α-MEM + 5% FBS + 50 μg/mL ascorbic acid + 1 μM dexamethasone + 3 mmol/L β-glycerophosphate. (i) α-MEM + 10% bovine calf serum + 16 ng/mL^−1^ dexamethasone + 10 nM β-glycerophosphate + 1.4 mM ascorbic acid. (j) α-MEM + 15% FBS + 100 U/mL penicillin + 100 μg/mL streptomycin + 200 mmol/L L-glutamine + 10 mmol/L ascorbic acid + 38 μg/mL dexamethasone. (k) α-MEM + 10% FBS + 1% penicillin/streptomycin + 0.1 μM dexamethasone + 10 μmol/L β-glycerophosphate + 50 μM ascorbic acid. (l) DMEM + 10% FBS + 1% penicillin/streptomycin + 10 mmol/L β-glycerophosphate + 50 μg/mL ascorbic acid. (m) DMEM + 10% FBS + 100 U/mL penicillin + 100 mg/mL streptomycin + 100 nM dexamethasone + 0.2 mM ascorbic acid + 10 nM β-glycerophosphate. (n) DMEM + 12% FBS + 100 μg/mL ascorbic acid + 100 μg/mL non-essential amino acids + 100 μg/mL penicillin/streptomycin + 100 nM dexamethasone + 0.2 mM 1-ascorbic acid-2-phophate. (o) DMEM + 10% FBS + 1% L- glutamine + 1% penicillin/streptomycin + 50 μM ascorbic acid + 10 nM 1.25(OH)D3 + 0.1 μM dexamethasone + 10 mM of β-glycerophosphate. (p) α-MEM + 10% FBS + 1% penicillin + 1% streptomycin + 0.2 mM ascorbic acid + 10mM β-glycerophosphate + 10 μM dexamethasone. NOM changing to OM: (q) MEM + 10% FBS + 100 U/mL of penicillin + 100 mg/mL non-essential amino acids + 50 μg/mL ascorbic acid/DMEM + 10% FBS + 100 U/mL of penicillin + 100 mg/mL non-essential amino acids + 50 μg/mL ascorbic acid + 10 mmol/L β-glycerophosphate + 0.1 μM dexamethasone. (r) DMEM + 10% FBS + 100 U/mL of penicillin/streptomycin + 100 μg/mL sodium ascorbate + 100 mg/mL non-essential amino acids/DMEM + 10% FBS + 100 U/mL of penicillin/streptomycin + 100 μg/mL sodium ascorbate + 100 mg/mL non-essential amino acids + 50 μM ascorbic acid + 10 mM β-glycerophosphate + 0.1 μM dexamethasone (NOM changes to OM on day 3. ALP assay was performed one day after medium changes. Alizarin Red staining and calcium deposition analysis were performed five days after medium changes). Legend: ADSCs—adipose-derived stem cells, ALP—alkaline phosphatase, BGLAP—osteocalcin, BMP-2—Bone Morphogenetic Protein-2, GMSCs—gingiva-derived stem cells, hBMSCs—human bone marrow stem cells, mBMSCs—murine bone marrow stem cells, mESCs—murine embryonic stem cells, OPN—osteopontin, OSX—osterix, RUNX2—Runt-related transcription factor 2, SIM—simvastatin, SMSCs—sinus maxillary stem cells, ↑—increase, ↓—decrease.

**Table 2:** The risk of bias in individual studies was assessed by the adapted Office of Health Assessment and Translation (OHAT) risk-of-bias tool. Ahmadi et al. (2022) [27], Chuang et al. (2015) [23], Huang et al. (2019) [24], Kupcsik et al. (2009) [25], Lee et al. (2019) [31], Ni et al. (2023) [16], Pagkalos et al. (2010) [29], Qiao et al. (2011) [28], Sabandal et al. (2022) [30], Shao et al. (2019) [22], Sun et al. (2009) [26], Tai et al. (2015) [15], Zhang et al. (2018) [21], and Zhou et al. (2010) [14]. After addressing all the questions, these scores were subsequently categorized into three tiers: Tier 1 (high quality), Tier 2 (moderate quality), or Tier 3 (low quality). Tier 1 is defined as having a “++” (definitely low) or “+” (probably low) risk of bias in all key domains, as well as a “++” (definitely low) or “+” (probably low) risk of bias for ≥50% of the other domains. Tier 2 comprises studies that do not meet the criteria for placement in either Tier 1 or Tier 3. Tier 3 represents a “- -” (definitely high) or “-” (probably high) risk of bias in all key domains AND “- -” (definitely high) or “-” (probably high) risk of bias for ≥50% of the other domains. Adapted with permission from Back, 2021 [37].

Bias Domains and Questions	Ahmadi et al. (2022) [27]	Chuang et al. (2015) [23]	Huang et al. (2019) [24]	Kupcsik et al. (2009) [25]	Lee et al. (2019) [31]	Ni et al. (2023) [16]	Pagkalos et al. (2010) [29]	Qiao et al. (2011) [28]	Sabandal et al. (2022) [30]	Shao et al. (2019) [22]	Sun et al. (2009) [26]	Tai et al., 2015 [15]	Zhang et al. (2018) [21]	Zhou et al. (2010) [14]
Selection Bias														
1. Was administered dose or exposure level adequately randomized?	++	++	++	++	++	++	++	++	++	++	++	++	++	++
2. Was allocation to study groups adequately concealed?	++	++	++	++	++	++	++	++	++	++	++	++	++	++
Performance Bias														
5. Were experimental conditions identical across study groups?	++	++	++	++	++	++	++	++	++	++	++	++	++	++
6. Were the research personnel and human subjects blinded to the study group during the study?	-	-	-	-	-	-	-	-	-	-	-	-	-	-
Attrition/Exclusion Bias														
7. Were outcome data complete without attrition or exclusion from the analysis?	++	++	++	++	++	++	++	++	++	++	-	++	++	+
8. Can we be confident in the exposure characterization?	++	++	++	++	++	++	++	+	++	++	++	++	++	++
9. Can we be confident in the outcome assessment?	++	++	++	++	++	++	++	++	++	++	-	++	++	+
Selective Reporting Bias														
10. Were all measured outcomes reported?	++	++	++	++	++	++	++	++	++	++	++	++	++	++
Other Sources of Bias														
11. Were there no other potential threats to internal validity (e.g., statistical methods were appropriate, and researchers adhered to the study protocol)?	++	++	++	++	++	++	++	++	++	++	-	++	++	+
Overall	tier 1	tier 1	tier 1	tier 1	tier 1	tier 1	tier 1	tier 1	tier 1	tier 1	tier 3	tier 1	tier 1	tier 1
	Answer Format													
++	Definitely low risk of bias: There is direct evidence of low-risk-of-bias practices. (May include a specific example of low-risk-of-bias practices).													
+	Probably low risk of bias: There is indirect evidence of low-risk-of-bias practices, OR it is deemed that deviations from low-risk-of-bias practices for these criteria during the study would not appreciably bias results, including consideration of direction and magnitude of bias.													
-	Probably high risk of bias: There is indirect evidence of high-risk-of-bias practices OR insufficient information (e.g., not reported or “NR”) provided about the relevant risk-of-bias practices.													
- -	Definitely high risk of bias: There is direct evidence of high-risk-of-bias practices. (May include a specific example of high-risk-of-bias practices).													

**Table 3:** Modified Grading of Recommendations, Assessment, Development, and Evaluation (GRADE) analysis.

Outcome	Object of Analysis	Relative Importance	Number of Studies	Certainty of the Evidence (GRADE)
Osteogenic Differentiation of Mesenchymal Cells				
Extracellular matrix mineralization	Von Kossa staining Alizarin Red staining Calcium content Phosphate content	Critical	14	⊕ ⊕ ⊕ ⊕ HIGH ^a,b^
ALP	Transcript ALP activity ALP staining	Critical	14	⊕ ⊕ ⊕ MODERATE ^a,b,c^
BMP-2	Transcript Protein expression	Critical	5	⊕ ⊕ ⊕ ⊕ HIGH ^a,b^
BMP-9	Transcript	Critical	1	⊕ ⊕ LOW ^d,e,f^
IBSP	Transcript	Critical	1	⊕ ⊕ LOW
RUNX2	Transcript Protein expression	Critical	7	⊕ ⊕ ⊕ MODERATE ^a,b,e^
COL1	Transcript Protein expression	Critical	5	⊕ ⊕ ⊕ ⊕ HIGH ^a,b^
BGLAP	Transcript Protein expression	Critical	8	⊕ ⊕ ⊕ ⊕ HIGH ^a,b^
OPN	Protein expression	Critical	1	⊕ ⊕ LOW ^d,e,f^
OSX	Transcript	Critical	3	⊕ ⊕ ⊕ MODERATE ^b,c,e^

GRADE Working Group grades of evidence: High certainty = we are very confident that the true effect lies close to that of the estimated effect. Moderate certainty = we are moderately confident in the effect estimate. The true effect is likely to be close to the estimate of the effect, but there is a possibility that it is substantially different. Low certainty = our confidence in the estimate is limited. The true effect may be substantially different from the estimate of the effect. Very low certainty = we have very little confidence in the effect estimate. The true effect is likely to be substantially different from the estimate of effect. Explanations: (^a^) Included studies presented methodological and statistical heterogeneity. (^b^) There is no standardization of methods, doses, or treatment regimens. (^c^) Studies from the same authors or conducted in the same institution presenting similar results. (^d^) Collectively, studies presented a high risk of bias. (^e^) Estimates were not sufficiently supported by the presented experiments. (^f^) The study presented a moderate risk of bias. Legend: ALP—alkaline phosphatase; BGLAP—osteocalcin; BMP-2—Bone Morphogenetic Protein-2; BMP-9—Bone Morphogenetic Protein-9; IBSP—Integrin Binding Sialoprotein; OPN—osteopontin; OSX—osterix; RUNX2—Runt-related transcription factor 2.

**Table 4:** Simvastatin concentrations used in the included studies. Ahmadi et al. (2022) [27], Chuang et al. (2015) [23], Huang et al. (2019) [24], Kupcsik et al. (2009) [25], Lee et al. (2019) [31], Ni et al. (2023) [16], Pagkalos et al. (2010) [29], Qiao et al. (2011) [28], Sabandal et al. (2022) [30], Shao et al. (2019) [22], Sun et al. (2009) [26], Tai et al. (2015) [15], Zhang et al. (2018) [21], and Zhou et al. (2010) [14].

Simvastatin Concentration	Reference
0.1 nM	[29]
0.3 nM	[21]
1 nM	[28,29]
10 nM	[14,22,28,29]
20 nM	[22]
100 nM	[14,16,24,27,28,29]
0.3 μM	[22]
0.5 μM	[23]
1 μM	[14,15,24,25,26,31]
5 μM	[25]
10 μM	[31]

## Abbreviations

The following abbreviations are used in this manuscript:

MDPI	Multidisciplinary Digital Publishing Institute
DOAJ	Directory of open access journals
TLA	Three-letter acronym
LD	Linear dichroism

## Appendix A

Data search strategy for the electronic databases (Embase, LILACS, LIVIVO, PubMed, SCOPUS, and Web of Science) and grey literature databases (Google Scholar and ProQuest). Adapted with permission from Back, 2021 [37].

**Database**	**Search Query**
PubMed	“Stem Cells” OR “Stem Cell” OR “Progenitor Cells” OR “Progenitor Cell” OR “Mother Cells” OR “Mother Cell” OR “Colony Forming Unit” OR “Colony Forming Units” OR “undifferentiated cells” OR Pericytes OR Pericyte OR “Rouget Cells” AND “Simvastatin” OR Zocor OR “MK 733” OR “MK733” OR Synvinolin OR “Hydroxymethylglutaryl CoA Reductase Inhibitors” OR “HMG CoA Reductase Inhibitors” OR “HMG CoA Statins” OR “Hydroxymethylglutaryl CoA Inhibitors” OR Statins OR “Hydroxymethylglutaryl Coenzyme A Inhibitors” AND “Osteogenesis” OR “bone formation” OR Ossification OR Osteoclastogenesis OR “osteoinduction” OR osteoinduct OR “Cell Differentiation” OR “Cell Differentiations” OR “Cell Growth Processes” OR “Bone Morphogenetic Proteins” OR “Bone Morphogenetic Protein” OR “Alkaline Phosphatase” OR Osteocalcin OR “Core Binding Factor Alpha 1 Subunit” OR “Runx2 Protein” AND “Osteogenesis” OR “bone formation” OR Ossification OR Ossifications OR Osteoclastogenesis OR osteoinduction OR osteoinducting OR osteoinductive OR osteoinductivity OR “Cell Differentiation” OR “Cell Differentiations” OR “Cell Growth Processes” OR “Bone Morphogenetic Proteins” OR “Bone Morphogenetic Protein” OR “Alkaline Phosphatase” OR Osteocalcin OR “Core Binding Factor Alpha 1 Subunit” OR “Runx2 Protein”
LILACS (English, Portuguese, and Spanish)	(“Stem Cells” OR “Stem Cell” OR “Progenitor Cells” OR “Progenitor Cell” OR “Mother Cells” OR “Mother Cell” OR “Colony Forming Unit” OR “Colony Forming Units” OR “Undifferentiated Cells” OR Pericytes OR Pericyte OR “Rouget Cells” OR “Células-Tronco” OR “Células Básicas” OR “Células Mãe” OR “Células Precursoras” OR “Células Primitivas” OR “Células Primordiais” OR “Células Progenitoras” OR “Células Tronco” OR “Unidades Formadoras de Colônias” OR “Pericitos” OR “Células de Rouget” OR “Células Madre” OR “Células Básicas” OR “Células Primitivas” OR “Células Primordiales” OR “Células Progenitoras” OR “Células Troncales” OR “Unidades que Forman Colonias”) AND (“Simvastatin” OR Zocor OR “MK 733” OR “MK733” OR Synvinolin OR “Hydroxymethylglutaryl CoA Reductase Inhibitors” OR “HMG CoA Reductase Inhibitors” OR “HMG CoA Statins” OR “Hydroxymethylglutaryl CoA Inhibitors” OR Statins OR “Hydroxymethylglutaryl Coenzyme A Inhibitors” OR “Sinvastatina” OR “Inibidores da Hidroximetilglutaril-CoA Redutases” OR “Estatinas” OR “Estatinas de HMG-CoA” OR “Inibidor de Hidroximetilglutaril-CoA Redutases” OR “Inibidores de HMG-CoA Redutases” OR “Simvastatina” OR “Inhibidores de Hidroximetilglutaril-CoA Reductasas” OR “Estatinas HMG-CoA” OR “Inhibidor de Hidroximetilglutaril-CoA Reductasas” OR “Inhibidor de Hidroximetilglutaril-CoA-Reductasa” OR “Inhibidor de las Hidroximetilglutaril-CoA Reductasas” OR “Inhibidores de HMG CoA Reductasa”) AND (Osteogenesis OR “bone formation” OR Ossification OR Osteoclastogenesis OR “osteoinduction” OR osteoinduct OR “Cell Differentiation” OR “Cell Differentiations” OR “Cell Growth Processes” OR “Bone Morphogenetic Proteins” OR “Bone Morphogenetic Protein” OR “Alkaline Phosphatase” OR Osteocalcin OR “Core Binding Factor Alpha 1 Subunit” OR “Runx2 Protein” OR Osteogênese OR “Formação dos Ossos” OR “Formação Óssea” OR Ossificação OR “Ossificação Endocondral” OR “Ossificação Fisiológica” OR Osteoclastogênese OR Osteoindução OR “Diferenciação Celular” OR “Processos de Crescimento Celular” OR “Processos de Crescimento da Célula” OR “Proteínas Morfogenéticas Ósseas” OR “Proteínas Morfogenéticas do Osso” OR “Proteínas Morfogênicas do Osso” OR “Proteínas Morfogênicas Ósseas” OR “Proteínas Ósseas Morfogenéticas” OR “Fosfatase Alcalina” OR Osteocalcina OR “Gla-Proteína Óssea” OR “Proteína de Ligação a Cálcio Dependente de Vitamina K” OR “Proteína de Ligação a Cálcio Vitamina K-Dependente” OR “Proteína Óssea Dependente de Vitamina K” OR “Proteína Óssea Vitamina K-Dependente” OR “Subunidade alfa 1 de Fator de Ligação ao Core” OR Osteogénesis OR “Formación del Hueso” OR Osificación OR “Osificación Endocondral” OR “Osificación Fisiológica” OR Osteoclastogénesis OR Osteoinducción OR “Diferenciación Celular” OR “Procesos de Crecimiento Celular” OR “Proteínas Morfogenéticas Óseas” OR “Proteínas Morfogenéticas de Hueso” OR “Proteínas Óseas Morfogenéticas” OR “Fosfatasa Alcalina” OR “Gla-Proteína Ósea” OR “Proteína Gla del Hueso” OR “Proteína de Unión a Calcio Dependiente de Vitamina K” OR “Proteína de Unión a Calcio Vitamina K-Dependiente” OR “Proteína Ósea Dependiente de Vitamina K” OR “Proteína Ósea Vitamina K-Dependiente” OR “Subunidad alfa 1 del Factor de Unión al Sitio Principal” OR “Subunidad alfa del CBF”)
SCOPUS	(TITLE-ABS-KEY(“Stem Cells” OR “Stem Cell” OR “Progenitor Cells” OR “Progenitor Cell” OR “Mother Cells” OR “Mother Cell” OR “Colony Forming Unit” OR “Colony Forming Units” OR “undifferentiated cells” OR Pericytes OR Pericyte OR “Rouget Cells”) AND TITLE-ABS-KEY(Simvastatin OR Zocor OR “MK 733” OR “MK733” OR Synvinolin OR “Hydroxymethylglutaryl CoA Reductase Inhibitors” OR “HMG CoA Reductase Inhibitors” OR “HMG CoA Statins” OR “Hydroxymethylglutaryl CoA Inhibitors” OR Statins OR “Hydroxymethylglutaryl Coenzyme A Inhibitors”) AND TITLE-ABS-KEY(Osteogenesis OR “bone formation” OR Ossification OR Osteoclastogenesis OR “osteoinduction” OR osteoinduct OR “Cell Differentiation” OR “Cell Differentiations” OR “Cell Growth Processes” OR “Bone Morphogenetic Proteins” OR “Bone Morphogenetic Protein” OR “Alkaline Phosphatase” OR Osteocalcin OR “Core Binding Factor Alpha 1 Subunit” OR “Runx2 Protein”)
EMBASE	(‘stem cells’/exp OR ‘stem cells’ OR ‘stem cell’/exp OR ‘stem cell’ OR ‘progenitor cells’ OR ‘progenitor cell’/exp OR ‘progenitor cell’ OR ‘mother cells’ OR ‘mother cell’/exp OR ‘mother cell’ OR ‘colony forming unit’/exp OR ‘colony forming unit’ OR ‘colony forming units’ OR ‘undifferentiated cells’ OR ‘pericytes’/exp OR pericytes OR ‘pericyte’/exp OR pericyte OR ‘rouget cells’) AND (‘simvastatin’/exp OR simvastatin OR ‘zocor’/exp OR zocor OR ‘mk 733’/exp OR ‘mk 733’ OR ‘mk733’/exp OR ‘mk733’ OR ‘synvinolin’/exp OR synvinolin OR ‘hydroxymethylglutaryl coa reductase inhibitors’/exp OR ‘hydroxymethylglutaryl coa reductase inhibitors’ OR ‘hmg coa reductase inhibitors’/exp OR ‘hmg coa reductase inhibitors’ OR ‘hmg coa statins’ OR ‘hydroxymethylglutaryl coa inhibitors’ OR ‘statins’/exp OR statins OR ‘hydroxymethylglutaryl coenzyme a inhibitors’) AND (‘osteogenesis’/exp OR osteogenesis OR ‘bone formation’/exp OR ‘bone formation’ OR ‘ossification’/exp OR ossification OR ossifications OR ‘osteoclastogenesis’/exp OR osteoclastogenesis OR ‘osteoinduction’/exp OR osteoinduction OR osteoinducting OR osteoinductive OR ‘osteoinductivity’/exp OR osteoinductivity OR ‘cell differentiation’/exp OR ‘cell differentiation’ OR ‘cell differentiations’ OR ‘cell growth processes’/exp OR ‘cell growth processes’ OR ‘bone morphogenetic proteins’/exp OR ‘bone morphogenetic proteins’ OR ‘bone morphogenetic protein’/exp OR ‘bone morphogenetic protein’ OR ‘alkaline phosphatase’/exp OR ‘alkaline phosphatase’ OR ‘osteocalcin’/exp OR osteocalcin OR ‘core binding factor alpha 1 subunit’/exp OR ‘core binding factor alpha 1 subunit’ OR ‘runx2 protein’/exp OR ‘runx2 protein’)
Web of Science	TS=(“Stem Cells” OR “Stem Cell” OR “Progenitor Cells” OR “Progenitor Cell” OR “Mother Cells” OR “Mother Cell” OR “Colony Forming Unit” OR “Colony Forming Units” OR “undifferentiated cells” OR Pericytes OR Pericyte OR “Rouget Cells”) AND TS=(Simvastatin OR Zocor OR “MK 733” OR “MK733” OR Synvinolin OR “Hydroxymethylglutaryl CoA Reductase Inhibitors” OR “HMG CoA Reductase Inhibitors” OR “HMG CoA Statins” OR “Hydroxymethylglutaryl CoA Inhibitors” OR Statins OR “Hydroxymethylglutaryl Coenzyme A Inhibitors”) AND TS=(Osteogenesis OR “bone formation” OR Ossification OR Osteoclastogenesis OR “osteoinduction” OR osteoinduct OR “Cell Differentiation” OR “Cell Differentiations” OR “Cell Growth Processes” OR “Bone Morphogenetic Proteins” OR “Bone Morphogenetic Protein” OR “Alkaline Phosphatase” OR Osteocalcin OR “Core Binding Factor Alpha 1 Subunit” OR “Runx2 Protein”)
ProQuest	noft(“Stem Cells” OR “Stem Cell” OR “Progenitor Cells” OR “Progenitor Cell” OR “Mother Cells” OR “Mother Cell” OR “Colony Forming Unit” OR “Colony Forming Units” OR “undifferentiated cells” OR Pericytes OR Pericyte OR “Rouget Cells”) AND (“Simvastatin” OR Zocor OR “MK 733” OR “MK733” OR Synvinolin OR “Hydroxymethylglutaryl CoA Reductase Inhibitors” OR “HMG CoA Reductase Inhibitors” OR “HMG CoA Statins” OR “Hydroxymethylglutaryl CoA Inhibitors” OR Statins OR “Hydroxymethylglutaryl Coenzyme A Inhibitors”) AND (Osteogenesis OR “bone formation” OR Ossification OR Osteoclastogenesis OR “osteoinduction” OR osteoinduct OR “Cell Differentiation” OR “Cell Differentiations” OR “Cell Growth Processes” OR “Bone Morphogenetic Proteins” OR “Bone Morphogenetic Protein” OR “Alkaline Phosphatase” OR Osteocalcin OR “Core Binding Factor Alpha 1 Subunit” OR “Runx2 Protein”)
Google Scholar	“Undifferentiated Cells” AND “Simvastatin” AND “Cell Differentiation” AND “Bone Formation”

## Appendix B. Articles Excluded and the Reasons for Exclusion (*n* = 63)

**Author (Year)**	**Reason for Exclusion**
Aly et al. (2018)	8
Arpornmaeklong et al. (2017)	2
Baadani et al. (2023)	2
Baek et al. (2005)	8
Biouki et al. (2019)	2
Chi et al. (2019)	7
Cui et al. (2013)	5
Feng et al. (2020)	7
Huang et al. (2014)	2
Huang et al. (2019)	5
Hungaro et al. (2017)	2
Janz et al. (2014)	8
Jin et al. (2011)	11
Kamada et al. (2017)	8
Kim et al. (2011)	1
Lai et al. (2018)	2
Lai et al. (2019)	1
Lee et al. (2016)	2
Lee et al. (2018)	2
Li et al. (2018)	2
Liu et al. (2009)	8
Liu et al. (2014)	2
Liu et al. (2014)	8
Liu et al. (2016)	8
Matos et al. (2015)	2
Niu et al. (2015)	8
Oh et al. (2021)	2
Park et al. (2012)	1
Pullisaar et al. (2014)	2
Qi et al. (2013)	2
Qing et al. (2019)	4
Rostami et al. (2020)	2
Samiei et al. (2016)	2
Simões et el. (2013)	2
Song et al. (2003)	8
Song et al. (2008)	8
Sukul et al. (2015)	2
Tai et al. (2010)	6
Tai et al. (2011)	6
Tai et al. (2013)	6
Wadagaki et al. (2011)	2
Wang et al. (2014)	2
Wang et al. (2016)	6
Wang et al. (2018)	2
Wang et al. (2020)	1
Wu et al. (2014)	4
Wu et al. (2020)	2
Wu et al. (2021)	2
Xiaolin et al. (2023)	11
Xu et al. (2015)	4
Xu et al. (2018)	5
Xue et al. (2019)	2
Yu et al. (2017)	2
Yuan et al. (2019)	2
Yun et al. (2013)	8
Zanette et al. (2015)	5
Zhang et al. (2015)	2
Zhang et al. (2018)	2
Zhang et al. (2018)	2
Zhang et al. (2019)	4
Zhao & Liu (2014)	7
Zheng et al. (2010)	1
Zijah et al. (2016)	2
Xiaolin et al. (2023)	11
Baadani et al. (2023)	2
Legend: (1) Studies investigating other cell types, such as differentiated cells. (2) Studies assessing drug or biomaterial associations that do not present separate data for simvastatin alone. (3) Studies that do not present an untreated control group with the same cell type. (4) Studies that do not present a control group without simvastatin treatment. (5) Studies that do not evaluate osteogenic differentiation. (6) Studies that are not in vitro (clinical studies, animal studies, conference abstracts, letters, pilot studies, review articles, case reports, protocols, short communications, personal opinions, posters, and book chapters). (7) Studies that do not specify the culture medium. (8) Studies that do not quantify alkaline phosphatase (ALP) or extracellular matrix mineralization for test and control groups. (9) Full text not available (book chapters, conference abstracts, expert opinions, letters, and literature reviews). (10) Duplicated data (e.g., dissertations/theses in which corresponding published articles were available). (11) Studies not published in the Latin alphabet. Adapted with permission from Back, 2021 [37].	
